# Satisfying Results of Primary Hip Arthroplasty in Patients With Hip Dysplasia at a Mean Followup of 20 Years

**DOI:** 10.1007/s11999-016-4998-6

**Published:** 2016-08-04

**Authors:** Ena Colo, Wim H. C. Rijnen, Jean W. M. Gardeniers, Albert van Kampen, B. Willem Schreurs

**Affiliations:** 1 Department of Orthopaedic Surgery, Radboud University Medical Center, Nijmegen, Netherlands; 2 Department of Orthopaedic Surgery 611, Radboud University Medical Center, PO Box 9101, 6500 HB Nijmegen, Netherlands

## Abstract

**Background:**

Developmental dysplasia of the hip (DDH) is a common cause of secondary osteoarthritis (OA) in younger patients, and when end-stage OA develops, a THA can provide a solution. Different options have been developed to reconstruct these defects, one of which is impaction bone grafting combined with a cemented cup. To determine the true value of a specific technique, it is important to evaluate patients at a long-term followup. As there are no long-term studies, to our knowledge, on THA in patients with DDH using impaction bone grafting with a cemented cup, we present the results of this technique at a mean of 15 years in patients with previous DDH.

**Questions/purposes:**

We wished to determine (1) the long-term probability of cup revision at a minimum followup of 15 years for cemented acetabular impaction bone grafting in patients with DDH; (2) the radiographic appearance of the bone graft and radiographic signs of implant loosening; and (3) the complications and pre- and postoperative Harris hip scores with cemented THA combined with impaction bone grafting in patients with previous DDH.

**Methods:**

Between January 1984 and December 1995 we performed 28 acetabular impaction bone grafting procedures for secondary OA believed to be caused by DDH in 22 patients; four patients died before 15 years, leaving 24 hips in 18 patients for retrospective analysis at a minimum of 15 years (mean, 20 years; range, 16–29 years). The diagnosis of DDH was made according to preoperative radiographs and intraoperative findings. All grades of dysplasia were included; five patients had Crowe Group I, eight had Group II, nine had Group III, and two had Group IV DDH. No patients were lost to followup. In all cases the acetabular defects were combined cavitary and segmental. Owing to the high number of deaths, we performed a competing-risk analysis to determine the probability of cup revision surgery.

**Results:**

The competing-risk analysis showed cumulative incidences at 15 and 20 years, with endpoint revision for any reason of 7% (95% CI, 0%–17%), whereas this was 4% (95% CI, 0%–11%) with endpoint revision of the cup for aseptic loosening. Three revision surgeries were performed. Two cup revisions were performed for aseptic loosening at 12 and 26 years. Another cup revision was performed owing to sciatic nerve problems at 2 years. A stable radiographic appearance of the graft was seen in 19 of the 25 unrevised hips. Four hips showed acetabular radiolucent lines and two showed acetabular osteolysis. None of the unrevised cups showed migration or radiographic failure. Postoperative complications included a pulmonary embolus and a superficial wound infection. The Harris hip score improved from 37 (range, 9–72) preoperatively to 83 (range, 42–99) at latest followup.

**Conclusions:**

Cemented primary THA with the use of impaction bone grafting shows satisfying long-term results in patients with previous DDH. For future research it is important to evaluate this technique in a larger cohort with a long-term followup. Other techniques also should be evaluated at long-term followup to be able to compare different techniques in this important and specific patient group.

**Level of Evidence:**

Level IV, therapeutic study.

## Introduction

Developmental dysplasia of the hip (DDH) is a common cause of secondary osteoarthritis (OA) in younger patients, and when end-stage OA develops, THA is a good surgical option [17]. However, THA in this patient group is a demanding procedure owing to the underlying acetabular bone stock defects, which hamper anatomic reconstruction. Therefore, these bone defects and the often-young age of these patients result in higher failure rates of their THAs relative to patients with primary OA [8, 33].

One option to try to improve cup survivorship in this setting is impaction bone grafting combined with a cemented cup. This technique was developed by Slooff [32] and was used primarily in hips with protrusio acetabuli and also for revision THA [28–30, 32]. Slooff [32] observed union of all grafts at an average followup of 2 years after primary THA in patients with protrusion. With revision THA, using the same technique, Schreurs et al. [28] reported an 87% survival rate for the cup at 20 years with an endpoint of aseptic loosening. We thought this technique might be an option in primary THA in patients with DDH to repair acetabular defects and possibly provide a durable solution in these difficult primary THAs. To the best of our knowledge, there are no studies that report the long-term outcomes of this technique in primary THA for patients with DDH. However, outcomes of other reconstruction methods have been reported at long-term followup for primary THA in this patient group [1, 11, 14, 18, 31, 35]. Abdel et al. [1] reported 66% survival at 20 years in patients with DDH and uncemented cups, who underwent reconstruction with a bulk femoral head autograft. Gill et al. [11] evaluated the use of reinforcement rings in 33 patients with DDH. They reported nine (10%) revisions at a mean followup of 11 years, of which six were attributable to infection. Comparison of our technique with these other techniques is important to be able to provide the most optimal care for this patient group.

Specifically, we sought to determine (1) the long-term probability of cup revision at a minimum followup of 15 years of cemented acetabular impaction bone grafting in patients with DDH; (2) the radiographic appearance of the bone graft and radiographic signs of implant loosening at long-term followup; and (3) the complications and pre- and postoperative Harris hip scores of cemented THA combined with impaction bone grafting in patients with previous DDH. In a previous report Somford et al. [34] presented the results of 28 THAs with a minimum followup of 10 years.

## Patients and Methods

We retrospectively studied all patients with secondary OA resulting from DDH, and who received a primary THA in one tertiary care institution between January 1984 and December 1995. We have always used cemented THAs, and in the case of acetabular defects, reconstruction with the use of impaction bone grafting and, if needed, a metal mesh. No other reconstruction techniques were used. All reconstructions were performed with impacted morselized bone grafts combined with a cemented THA. The original series consisted of 28 hips in 22 patients (Table 1); four patients died (four hips) before postoperative year 15; their data are included. Subsequently, we reviewed 24 hips in 18 patients with a minimum followup of 15 years (mean, 20 years; range, 16–29 years). Two patients (three hips) died at 16, 16, and 20 years postoperatively. In all cases, death was unrelated to the hip surgery and no reoperations had been performed at the time of death. No patients were lost to followup.

**Table 1:** Patient details

Variable	Number of hips (number of patients)	Results
Number of procedures and patients	28 (22)	No patients lost to followup
Deaths	7 (6)	Deaths at 3, 6, 10, 13, 16, 16, and 20 years without reoperation
Available with minimum followup of 15 years	24 (18)	4 patients died before postoperative year 15
Revisions	3 (3)	One cup revision resulting from sciatic nerve problems at 2 years One total hip revision resulting from aseptic loosening at 12 years One cup revision resulting from aseptic loosening at 26 years

The study group consisted of 17 females and one male with a mean age at surgery of 48 years (range, 26–74 years). Thirteen (54%) operations were performed in patients younger than 50 years and 22 (92%) were done in patients younger than 60 years. Six patients had bilateral THAs, and 12 procedures were right-sided. Two patients (four hips) were unable to visit the outpatient clinic and radiographs were not taken owing to the patients’ age or poor health status, which was unrelated to the hip surgery. All the other patients who had not undergone revision surgery or had not died were seen in the outpatient clinic during the past 5 years. However, these patients were contacted and the questionnaires were completed by phone. The latest radiographic followups of these patients were at 12, 13, 16, and 16 years postoperatively.

In all cases the acetabular defects were combined cavitary and segmental (Type 3) according to the American Academy of Orthopaedic Surgeons classification [4]. The severity of dysplasia was graded according to the Crowe et al. [3] and Eftekhar classifications [7]. Five hips had Crowe Group I, eight had Group II, nine had Group III, and two had Group IV dysplasia. For the Eftekhar classification, seven hips were Type A, 12 were Type B, and five were Type C.

A detailed description of the surgical technique was described previously [34].

In 23 hips an autograft from the femoral head was used and a combination of an auto- and allograft, obtained from the bone bank, was used in one. Nineteen Elite cups (DePuy, Leeds, UK) and nine Müller cups (Sulzer, Winterthur, Switzerland) were inserted.

During that time, postoperative management consisted of systemic antibiotics for 5 days, indomethacin for 5 days for prevention of heterotopic ossifications, and anticoagulation for 3 months. Passive movement was started at 24 hours. Partial weightbearing was started at 3 weeks in five patients and at 6 weeks in 19 patients. Full weightbearing was allowed at 3 months postoperatively. For clinical evaluation, the pre- and postoperative Harris hip score was used [13].

Radiographic followup was done using AP and lateral views of the hip and was scored by two of the authors (EC, BWS) and classified on a consensus basis. The following parameters were scored: graft incorporation, height of the center of rotation, radiolucent lines (> 2 mm wide), or osteolysis in one of the three zones of DeLee and Charnley [6], and migration of the cup. Radiographic failure was defined as the presence of radiolucent lines in the three zones of DeLee and Charnley or migration of 5 mm or more in any direction.

Competing-risk analyses were performed to determine the probability of revision of the acetabular component in the presence of the competing event of death with endpoints of revision for any reason and aseptic loosening.

## Results

The cumulative failure rate of the acetabular component, with use of the competing-risk analysis, was 7% (95% CI, 0%–17%) with the endpoint revision for any reason at 15 and 20 years, and 4% (95% CI, 0%–11%) at 15 and 20 years with the endpoint aseptic loosening (Table 2). One additional revision has been performed since the initial report by Somford et al. [34]. Thus, in total, three revisions were performed during followup. The first patient to undergo revision surgery had Crowe Group III dysplasia and had sciatic nerve palsy develop postoperatively after reconstruction of a high hip center. A cup revision was performed to recreate a higher hip center to release the sciatic nerve. The second patient (with Crowe Group I dysplasia) showed progressive radiolucent lines in all three acetabular zones, which led to revision resulting from aseptic loosening. Both components were revised 12 years after the primary procedure. The last patient (with Crowe Group III dysplasia) showed progressive migration of the cup, and cup revision owing to aseptic loosening was performed 26 years after the primary procedure.

**Table 2:** Cumulative failure rates

Endpoint	Followup (years)	Survival percentage (95% CI)
Revision for any reason	15	7% (0%–17%)
	20	7% (0%–17%)
Revision for aseptic loosening	15	4% (0%–11%)
	20	4% (0%–11%)

**Fig. 1A–C:**
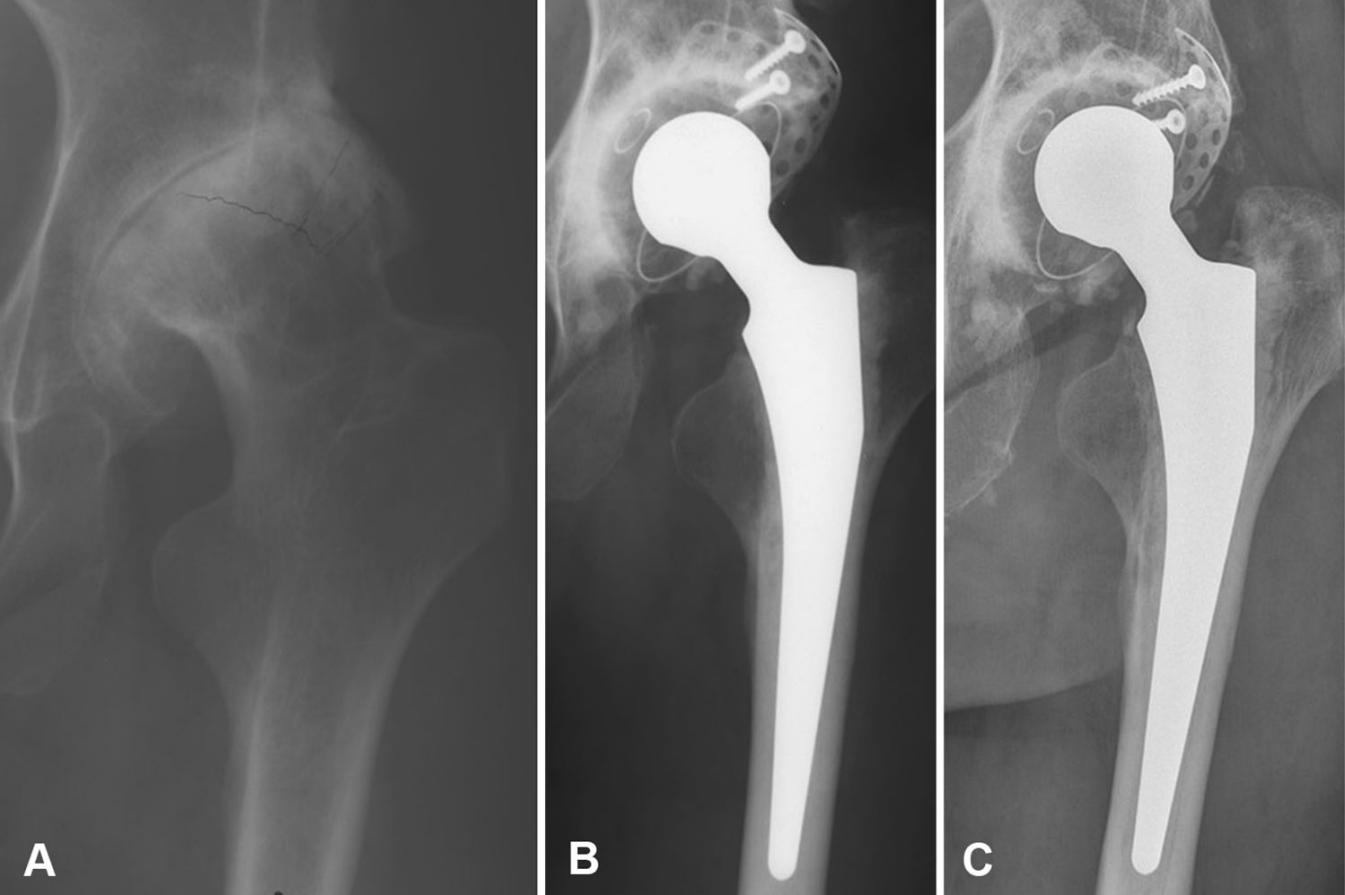
(**A**) A preoperative radiograph, (**B**) immediate postoperative radiograph after a THA with a cemented cup, lateral metal mesh, and impaction bone grafting, and (**C**) after followup of 25 years are shown. The THA prosthesis is still in situ and the patient, a 49-year-old woman with Crowe Group II dysplasia on the left side, is without complaints and has good function. A radiolucency is visible in acetabular Zone 3, but has remained stable during the past 10 years.

A stable radiographic appearance of the graft and cup was seen in 19 of the 25 unrevised hips. Four hips showed radiolucent lines, three hips in acetabular Zone III (Fig. 1) of which two were progressive. The other hip had progressive radiolucency in Zones I and II. Two hips showed acetabular osteolysis; in one hip (followup, 18 years), this previously was scored as a radiolucent line but progressed to an osteolytic area. The other hip (followup, 29 years) showed osteolysis in Zone III, which remained stable during the past 5 years. None of the unrevised cups showed migration or radiographic failure. Additionally, none of the femoral components showed radiographic loosening. The mean height of the center of rotation was 31 mm (range, 19–50 mm), of which three hips had a center of rotation of 35 mm or greater.

During followup, no dislocations or infections of the prostheses occurred. Three patients experienced a complication. One patient, as mentioned above, had sciatic nerve palsy and underwent cup revision for this reason. One patient had a pulmonary embolus postoperatively and was treated with intravenous heparin, and another patient had a superficial wound infection and was treated with antibiotics. The infection resolved and the prosthesis is still in situ. The mean Harris hip score for the patients without revision surgery with a minimum followup of 15 years was 37 (range, 9–72) preoperatively and improved to 83 (range, 42–99) at latest followup.

## Discussion

Secondary OA of the hip attributable to DDH is a common indication for THA, especially in younger patients [17]. However, this specific patient group is challenging owing to the acetabular defects that usually come along with the dysplasia. Numerous studies have reported reconstruction methods for acetabular defects using various operative techniques [12, 15, 23, 24, 36]. Comparisons with other techniques and the presence of long-term followup reports [13, 16, 24, 25, 36] are important to evaluate the durability of a technique or implant. To the best of our knowledge, there are no other studies presenting 20-year results of this technique in patients with DDH. Our study showed a 7% probability of cup revision surgery with revision for any reason as the endpoint at 15 years and 20 years in patients with THA after previous DDH. A previous study [34] showed a survival of 96% at 10 years with revision for any reason as the endpoint. Limitations of the current study include the small number of patients and various grades of dysplasia were included, which could cause bias as the number of hips with high grades of dysplasia is low which makes it less reliable to draw conclusions for patients with severely dysplastic hips. Although the number of patients with severely affected hips is low, we decided to include all patients during this period. In addition, two patients (four hips) were not able to visit the outpatient clinic or have their radiographs taken. However, we contacted these patients and obtained information regarding their hip status. All other patients were seen in the outpatient clinic during the past 5 years. Finally, in some cases, the radiographic assessment can be hampered by the reconstruction material, cement, and prosthesis. We tried to minimalize this assessment bias by reviewing all subsequent radiographs of each patient and discussing the difficult radiographs until a consensus was reached.

Several studies on THA in patients with previous DDH have been reported, using different reconstruction methods (Table 3) [1, 2, 9, 11, 16, 18, 25]. However, to our knowledge, there are no studies using cemented cups with impaction bone grafting reporting a minimum followup of 15 years. There are other study groups who have reported reasonable results for uncemented implants with the use of impaction bone grafting [20, 22, 26, 27]. Lee and Nam [20] reported a satisfying 12-year survival rate of 96% with uncemented cups and allograft impaction bone grafting in revision THA. However, these studies did not stratify for the diagnosis of DDH. We have performed only one additional revision since the previous report by Somford et al. [34], and that revision was 26 years after the primary THA and attributable to aseptic loosening. The first revision occurred after 2 years in a patient with a sciatic nerve palsy. A cup revision with more-proximal placement of the cup was performed to release the sciatic nerve. It is a known problem that patients with THA with high grades of dysplasia are at risk for sciatic nerve problems owing to a change in the height of the center of rotation [5, 26]. We now shorten the femur in these patients to avoid lengthening greater than 3 cm and subsequently reduce the risk of neurologic palsies [19, 21]. A rationale behind the use of impaction bone grafting is that it possibly can improve the bone stock and therefore future revisions may be facilitated by the graft used at primary THA; however, future research will need to determine to what degree this might be true. In the current study, there were few revisions and no comparison group, therefore it is not possible to comment on this potentially important endpoint.

**Table 3:** Outcomes of different reconstruction methods

Study	Number of hips (patients)	Classification of dysplasia	Mean followup, years (range)	Fixation method	Reconstruction method	Number and reasons of acetabular component revisions	Acetabular component survival	Radiographic appearance
Gill et al. [11] 1998	87 (70)	Crowe Group II, 11; Group III, 65; Group IV, 11	9 (5–15)	Cemented	Reinforcement ring	Cup revisions, 8; aseptic loosening, 2; septic revisions, 6	Not available	Definitely loose, 5 cups; probably loose, 2 cups; possibly loose, 13 cups; migration, 6 cups; radiolucent lines, 15 cups
Rozkydal et al. [25] 2005	43 (43)	Crowe Group I, 6; Group II, 31; Group III, 3; Group IV, 3	10 (9–11)	Uncemented	Structural femoral head autograft	No revisions	Any reason, 100% at 10 years; any reason with radiographic loosening, 88% at 10 years	No failures of bone graft; osteolysis, 1 cup; radiolucent line, 1 cup
Chougle et al. [2] 2006	292 (206)	Crowe Group I, 161; Group 2, 78; Group III, 27; Group IV, 26	16 (2–31)	Cemented	Structural bone grafting in 48 hips	Aseptic loosening, 68; septic revisions, 4; revisions in the bone grafting group, 4	Aseptic loosening, 63% at 20 years; radiographic failure, 62% at 20 years	Complete demarcation, 16 cups; bone grafting group, 37 unions of the graft
Eskelinen et al. [9] 2006	68 (56)	High dislocations, 68	12 (9–15)	Uncemented	Bulk femoral head autograft (unknown how many cases)	Cup revisions, 26; aseptic loosening, 12; failed liners/wear, 14	Press-fit cup (n = 59), any reason, 88% at 10 years; aseptic loosening, 95% at 10 years	Migration and radiolucent lines, 1 cup; periacetabular osteolysis., 2 cups
Karachalios et al. [18] 2013	61 (44)	Low dislocations, 25; high dislocations, 36	24 (20–32)	Cemented	Cotyloplasty	Cup revisions, 29; aseptic loosening, 28; dislocation, 1	Aseptic loosening, 56% at 23 years	Radiographic loosening, 3 cups
Abdel et al. [1] 2014	35 (29)	Crowe Group I, 7; Group II, 5; Group III, 19; Group IV, 4		Uncemented	Bulk femoral head autograft	Cup revisions, 12; shell/liner revisions, 9; aseptic loosening, 1; fracture of liner, 1; instability, 1	Aseptic loosening, 66% at 20 years	No identifiable resorption of the graft supporting the component
Iwase et al. [16] 2016	40 (38)	Crowe Group I, 13; Group II, 15; Group III, 11; Group IV, 1	8 (3–10)	Cemented	Impaction bone grafting with a metal mesh	Cup revision, septic revision, 1	Any reason, 100% at 8 years; aseptic loosening, 100% at 8 years	No radiolucent lines or loosening
Current study	24 (18)	Crowe Group I, 5; Group II, 8; Group III, 9; Group IV, 2	20 (16–29)	Cemented	Impaction bone grafting	Cup revisions, 3; femoral nerve palsy, 1; aseptic loosening, 2	Cumulative incidence: any reason, 7% at 20 years; aseptic loosening, 4% at 20 years	Radiolucent lines, 4 cups; osteolysis, 2 cups

Gill et al. [11] reported a high rate of cups that showed signs of radiographic loosening. Iwase et al. [16] applied impaction bone grafting combined with cemented cups and a metal mesh and did not observe radiolucent lines or loosening of the cup in their patients (Table 3). In total in our patients, four hips showed radiolucent lines in one or two acetabular zones. However, none of these hips showed radiolucent lines in all three zones. Additionally, even after a followup of 15 years or more these hips showed no signs of migration or changes in cup position, which makes complications or loosening in the near future less likely [10]. Another two hips showed osteolysis around the cup and, although in one hip the osteolysis remained stable for the past 5 years, these patients need to be monitored closely for further progression of the osteolysis.

Regarding clinical outcomes, the Harris hip score at latest followup improved compared with the preoperative score and is acceptable after such a long followup. Compared with the report by Somford et al. [34], the Harris hip score remained consistent and satisfactory with time. Abdel et al. [1] and Eskelinen et al. [9] reported comparable pre- and postoperative Harris hip outcome scores with the use of bulk femoral head autografts.

We found satisfactory long-term results of THA combined with impaction bone grafting and a cemented cup in patients with previous developmental dysplasia. Even after a mean followup of 20 years, the probability of revision surgery remains 7% and no radiographic failures were detected. Although in our study, this technique showed promising results, studies evaluating this technique in independent centers with larger cohorts are necessary to evaluate its true value in patients with DDH. In addition, other techniques used for these patients should be reported, with large cohorts and longer followup.
